# Facile and Sensitive Acetylene Black-Based Electrochemical Sensor for the Detection of Imatinib

**DOI:** 10.1155/2023/3228470

**Published:** 2023-11-30

**Authors:** Shun Li, Qingwu Tian, Xuanming Xu, Chao Xuan, Xiaomin Yang, Shukai Sun, Tingting Zhou

**Affiliations:** Department of Clinical Laboratory, The Affiliated Hospital of Qingdao University, #No. 1677, Wutaishan Road, Qingdao 266000, Shandong, China

## Abstract

A facile and sensitive electrochemical sensor for determining imatinib (IMA) was constructed by modifying a glassy carbon electrode (GCE) with a nanocarbon material, acetylene black (AB). The electrochemical behavior of IMA on the prepared GCE/AB was studied using electrochemical techniques, namely, differential pulse voltammetry (DPV) and electrochemical impedance spectroscopy. The direct determination of IMA by the GCE/AB sensor was accomplished using DPV under optimized conditions. The method verification showed that the oxidation peak current was proportional to the concentrations of IMA in the linear ranges of 0.01–0.5 and 0.5–4 *μ*M, with correlation coefficients of 0.9856 and 0.9946, respectively. The limit of detection of the GCE/AB sensor was 0.15 nM. Moreover, the GCE/AB sensor showed good precision and accuracy. Finally, the GCE/AB sensor was successfully applied to determine IMA in human serum samples, and the recoveries were satisfactory.

## 1. Introduction

Imatinib (IMA) is a tyrosine kinase inhibitor for the treatment of cancers, such as chronic myeloid leukemia [1], gastrointestinal stromal tumor [2], and dermatofibrosarcoma protuberans [3]. Reportedly, the molecular and cytogenetic response to IMA is related to the low plasma concentrations in patients. Therefore, IMA monitoring is necessary for clinical treatment [4]. Thus far, several analytical techniques have been applied to the detection of IMA, such as electrophoresis [5], high-performance liquid chromatography [6], and UV-vis spectroscopy [7]. However, due to the expensive equipment, long analysis process, and complicated operation steps of the existing techniques, an economical, fast, simple, and efficient method for IMA determination is urgently needed. The electrochemical method is a potential analytical technique for the detection of IMA and has attracted great attention because it provides several advantages, such as short analysis time, easy sample pretreatment procedures, and suitability for real-time detection [8]. According to previous research, an unmodified glassy carbon electrode (GCE) fails to provide a satisfactory effect for the detection of IMA, while modified GCE obtained indispensable adsorption and catalytic properties [9]. Acetylene black (AB) is a type of nanocarbon material formed by acetylene combustion in air under pressure [10], which has attracted tremendous attention because of its extraordinary conductivity and chemical stability [11], electrocatalytic properties [12], and adsorption ability [13], thereby promoting its further application in the field of electrochemical analysis. For instance, AB-modified electrodes exhibit superior electrochemical properties in measuring various substances, such as erythromycin [14], methotrexate [15], topotecan [16], methimazole [17], and chrysophanol [18]. To our knowledge, AB has never been used for the detection of IMA. Thus, AB is applied in this research to modify GCE for the fabrication of an electrochemical sensor (GCE/AB), which is applied for determining IMA in serum.

## 2. Reagents and Materials

IMA was supplied by Shanghai Aladdin Biochemical Technology Co., Ltd. AB, multiwalled carbon nanotubes (MWCNTs), graphene, amino carbon tubes, carboxyl carbon tubes, and hydroxyl carbon nanotubes were supplied by Shanghai Brinway Biotechnology Co., Ltd. Folic acid, glycine, vitamin C, citric acid, and glucose were supplied by Beijing Solarbio Technology Co., Ltd. Methanol, anhydrous ethanol, potassium ferrocyanide (K_4_[Fe(CN)_6_]), ferric chloride (FeCl_3_), hydrochloric acid (HCl), sodium hydroxide (NaOH), potassium chloride (KCl), sodium chloride (NaCl), zinc sulfate (ZnSO_4_), sodium dihydrogen phosphate dihydrate (NaH_2_PO_4_•2H_2_O), and disodium hydrogen phosphate dodecahydrate (Na_2_HPO_4_•12H_2_O) were purchased from Shanghai Sinopharm Chemical Reagent Co., Ltd. Gamma aluminum polishing powder (0.05 micron) was supplied by Wuhan Gaoss Union Science and Technology Ltd.

## 3. Instruments and Equipment

An electrochemical workstation (CHI660e) was obtained from Shanghai Chenhua Instrument Co., Ltd. The three-electrode system consists of the GCE/AB, the saturated calomel electrode, and the platinum electrode (Wuhan Gaoss Union Science and Technology Ltd.). A constant-temperature magnetic stirrer was purchased from Tianjin Sidis Experimental, an analysis instrument manufacturer. A pH meter (PHS-3E) was obtained from Shanghai INESA Scientific Instrument Co., Ltd. A CNC ultrasonic cleaner (KQ-50DB) was obtained from Kunshan Ultrasound Instrument Co., Ltd. Pipettes and a centrifuge were obtained from the German company Eppendorf. A pure water machine was obtained from Beijing Shuangfeng Zhongbang Technology Development Co., Ltd. A vortex mixer was obtained from Jiangsu Tianli Instrument Co., Ltd. A precision electronic balance was obtained from Sedis, Germany. A hard infrared light bulb was obtained from Shenzhen Anderson Lighting Co., Ltd. A refrigerated freezer (BCD-209 HFA) was obtained from Qingdao Aucma Co., Ltd.

## 4. Solution Preparation

The IMA solution (0.1 mM) was prepared by dissolving IMA in methanol. A phosphate buffer saline (PBS, 0.1 M) of different pH values was prepared with Na_2_HPO_4_•12H_2_O and NaH_2_PO_4_•2H_2_O, and the pH of the PBS was adjusted with the HCl and NaOH solutions. Then, 2.0 mg of AB was added to 2.0 mL of pure water, which was sonicated and dispersed for 1 h to obtain a finely evenly distributed black suspension (AB suspension). A redox probe containing 5 mM of [Fe(CN)_6_]3/4(1 : 1) and 0.1 M of KCl was prepared by dissolving potassium ferrocyanide (K_4_Fe(CN)_6_), potassium ferricyanide (K_3_Fe(CN)_6_), and potassium chloride (KCl) in pure water.

## 5. Electrode Preparation

First, the GCE surface was polished with gamma aluminum polishing powder. Subsequently, the electrode was washed sequentially with an ultrasonic washer in absolute ethanol and pure water for 5 min until the electrode surface was smooth as a mirror. Finally, 4.5 *μ*L of the AB suspension droplet was applied to the GCE surface and dried under an infrared bulb to obtain the GCE/AB.

## 6. Sample Preparation

First, 0.5 mL of human serum samples, which were placed at room temperature, was added into Eppendorf tubes, to which 1 mL of methanol was added. After an even oscillation on the vortex mixer, the samples were centrifuged at 10,000 rpm for 15 min. The supernatant was collected using a syringe and filtered through a 0.22 *μ*m microwell filter to obtain the filtrate, that is, the purified serum. A 20 *μ*L purified sample was added to the PBS (10 mL, 0.1 M, and pH 7.0) with specific IMA concentrations (0.1, 1, and 2 *µ*M) and finally detected by the GCE/AB electrochemical sensor.

## 7. Detection Method

PBS with a pH of 7.0 (10 mL) was selected as the reaction medium, to which 50 *μ*L of IMA solution (0.1 mM) was added. The PBS was stirred for 4 min, while the GCE/AB was enriched with IMA. After IMA enrichment, the differential pulse voltammetry (DPV) curves with the potential from 0.1 V to 0.9 V were recorded, and the scan rate, the pulse width, and the pulse amplitude were 20 mVs^−1^, 40 ms, and 50 mV, respectively. Electrochemical impedance spectroscopy (EIS) was applied in the redox probe in the frequency range of 0.01 Hz–100,000 Hz.

## 8. Results and Discussion

### 8.1. Fabrication and Characterization of the Sensor

**Figure 1:**
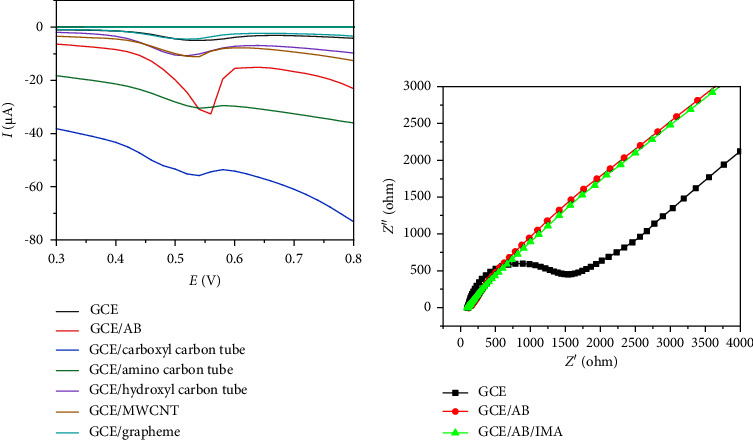
(a) Effects of different types of nanocarbon materials on the detection of 0.5 *μ*M of IMA. (b) EIS of bare GCE, GCE/AB, and GCE/AB/IMA in the redox probe of 0.1 M of KCl solution and 5 mM of [Fe(CN)_6_]^−3^/^−4^ (1 : 1).

A series of detection experiments using DPV to select the best electrochemical material for adsorbing and catalyzing IMA were performed. As shown in Figure 1(a), the oxidation peak current of IMA appeared at the potential of 0.560 V, but no reduction peak was observed in the reverse scan, indicating that the reaction of IMA on this electrochemical sensor was pure oxidation. After GCE modification, the oxidation peak current of IMA was improved by different materials. The AB-modified electrode (GCE/AB) performed with the highest oxidation peak current, indicating that the GCE/AB possesses the best adsorption and catalytic properties among the material-modified electrodes. This result demonstrates the feasibility of this research, again proving the high specific surface area, strong adsorption capacity, and excellent conductivity of AB [10]. Therefore, we selected AB as the electrode modification material for this experiment.  Figure 1(b) presents the interfacial characteristics of bare and differently modified GCE via EIS [19]. In the Nyquist plot of the EIS, the broader the semicircle curve of the plot is, the greater the resistance is [20]. The electron-transfer resistance at the electrode surface can be quantified by the diameter of the semicircle [21]. The diameter sizes followed the order GCE > GCE/AB/IMA > GCE/AB. A broad semicircle was monitored in the Nyquist plot for the bare electrode, indicating that the bare GCE faced high resistance. A narrow semicircle was observed for the GCE/AB, indicating a low resistance. The GCE/AB had a lower electron-transfer resistance than the bare GCE, confirming the successful construction of the GCE/AB-based electrochemical sensor. Moreover, the GCE/AB had a larger semicircle after IMA adsorption, indicating that the IMA adsorption on the GCE/AB surface increased the electron-transfer resistance. In addition, the Nyquist plot of the bare GCE showed a relatively large semicircle with an Rct of 1760 Ω. After the modification of the GCE with AB, the value of Rct decreased to 199 Ω, which confirmed that the electron-transfer property of AB/GCE improved.

**Figure 2:**
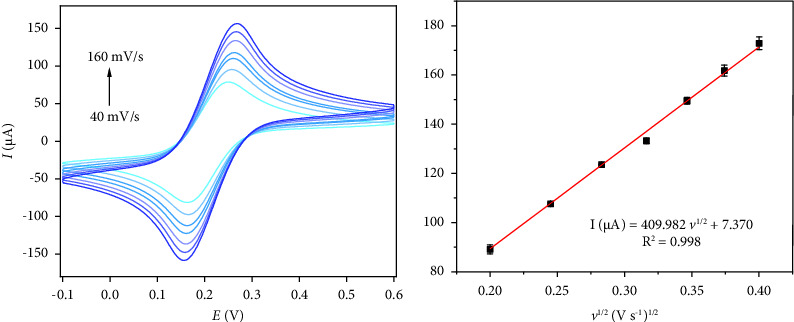
CVs (a) of AB/GCE in the presence of 0.25 mM of [Fe(CN)_6_]^3−^ solution in aqueous 0.1 M of KCl at various scan rates (40–160 mV/s). The plot (b) of peak currents vs. *υ*^1/2^.

The electrochemically active surface areas of the bare GCE and modified electrodes were calculated using CV at various scanning rates (40–160 mV s^−1^) in a solution containing 5.0 mM of [Fe(CN)6]3−/4− and 0.1 M of KCl. The efficacy of the embedded sensors was analyzed according to the Randles–Sevcik equation at 25°C [22]:

(1) $$\begin{array}{c} I_{P\;}=269000\;n^{3/2}{\;A\;D}^{1/2}{C\;v}^{1/2}, \end{array}$$

where *I*_*p*_ is the peak current, *n* is the electron-transfer number in the process (*n* = 1), *D* is the diffusion coefficient (7.6 × 10−6 cm^2^/s), A is the electrode surface area (cm^2^), C is the [Fe(CN)6]3−/4− concentration (mol/cm^3^), and *v* is the scanning rate (V/s). As shown in Figure 2, the slopes from the plot of current against the scanning rate square root were used for the calculation of the electroactive surface areas, and A values were 0.05 and 0.11 cm^2^ for the surfaces of bare GCE and AB/GCE, respectively. So AB could be reinforced during electroanalysis because of an impressive elevation in its electroactive surface area.

### 8.2. Optimization

#### 8.2.1. Effect of the Volume of AB Dispersion

**Figure 3:**
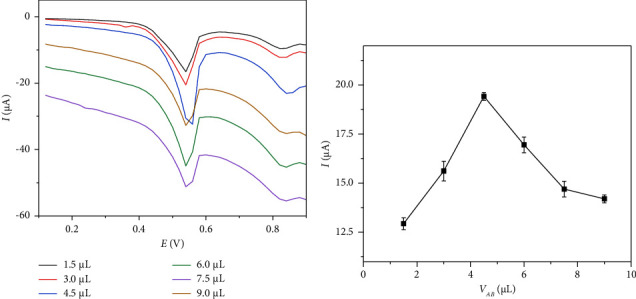
Effect of the AB dispersion solution's volume on the detection of 0.5 *μ*M of IMA in PBS (10 mL, 0.1 M, and pH 7.0) by the GCE/AB sensor.

Figure 3(a) presents the DPV curves of IMA detected by different volumes of the GCE/AB. Figure 3(b) shows that the oxidation peak current values of IMA on the GCE/AB sensor increased significantly as the volume of the AB dispersion increased from 1.5 *μ*L to 4.5 *μ*L, probably because the greater the amount of modified AB is, the greater the adsorption amount of IMA on the electrode surface is. When the volume of AB dispersion further increased to 8.0 *μ*L, the oxidation peak current values of IMA gradually decreased, probably because excessive AB on the modified electrode hinders the rate of electron transfer. Thus, the optimal volume of AB dispersion is 4.5 *μ*L, which was employed in further experiments.

#### 8.2.2. Effect of Enrichment Time

**Figure 4:**
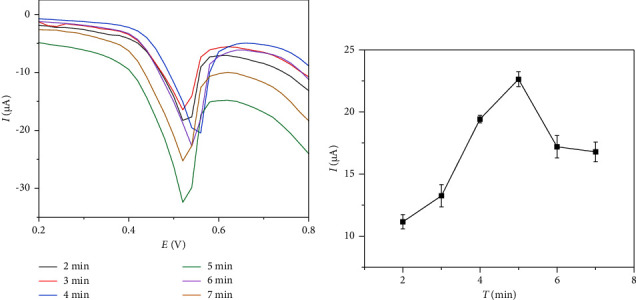
Effect of enrichment time on the detection of 0.5 *μ*M of IMA in PBS (10 mL, 0.1 M, and pH 7.0) by the GCE/AB sensor.

The effect of the IMA enrichment time on the detection was examined from 2 min to 7 min, and the DPV curves are displayed in Figure 4(a). As shown in Figure 4(b), the oxidation peak currents before 5 min were positively correlated with the enrichment time because the longer the enrichment time, the higher the amount of IMA adsorbed on the GCE/AB surface, but a negative correlation was observed thereafter. This finding indicates that the adsorption equilibrium of IMA on the electrode surface was achieved at 5 min, and excessive IMA could hinder the rate of electron transfer. The results show the highest IMA sensitivity and adsorption efficiency at the enrichment time of 5 min, which was employed in further experiments.

#### 8.2.3. Effects of Buffer pH and DPV Parameters

**Figure 5:**
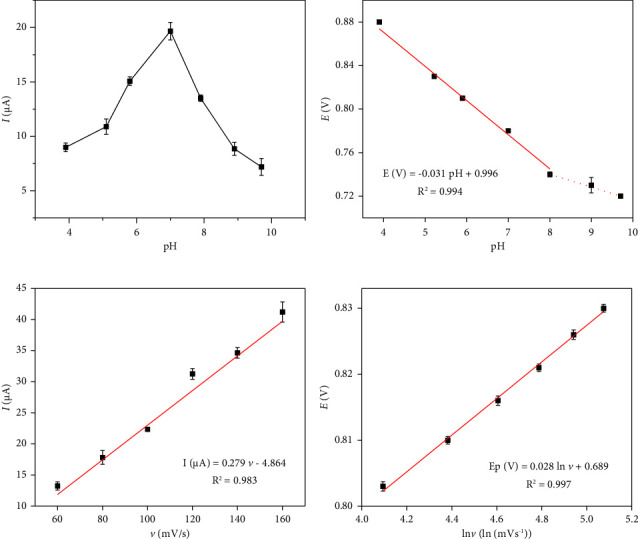
(a) Effects of buffer pH on the peak current of IMA by the GCE/AB sensor. (b) Potential diagram of different pH values in the presence of 0.5 *μ*M of IMA in PBS solution. (c) Corresponding plot of current vs. scanning rate (60–160 mV/s) in the presence of 0.5 *μ*M of IMA in PBS solution. (d) Linear relationship between *E*(*V*) and the logarithm of the scanning rate.

The effect of the buffer pH on the peak current of IMA was investigated by changing the pH value from 3.9 to 9.7. As shown in Figure 5(a), the peak current increased significantly from 3.9 to 7.0 and decreased in the pH range of 7.0–9.7. Thus, 7.0 is the optimal pH level. In addition to the buffer pH, the effects of DPV parameters on the results should be considered. The main DPV technique parameters, including the pulse width, scan rate, and pulse amplitude, were studied in the ranges of 20–100 ms, 10–30 mVs^−1^, and 20–200 mV, respectively. The results show that the peak pattern was the best when the pulse width, the scan rate, and the pulse amplitude were 40 ms, 20 mVs^−1^, and 50 mV, respectively.

### 8.3. Electrochemical Mechanism of IMA on the AB/GCE

The electrochemical response of IMA on the prepared electrode at different scanning rates was investigated to further explore its reaction mechanism. Figure 5(a) shows that the peak oxidation potential of IMA moved negatively as pH increased from 3.9 to 9.7. As can be seen in Figure 5(b), the pH value was linearly related to the oxidation peak potential of IMA, and its regression equation was EP (V) = −0.031pH + 0.996 (*R*^2^ = 0.994). The slope value of −32.13 mV was close to half of the theoretical value of Nernst's equation (−59 mV), which means that the number of protons involved in the IMA oxidation reaction was half of the electrons [23].

**Scheme 1:**
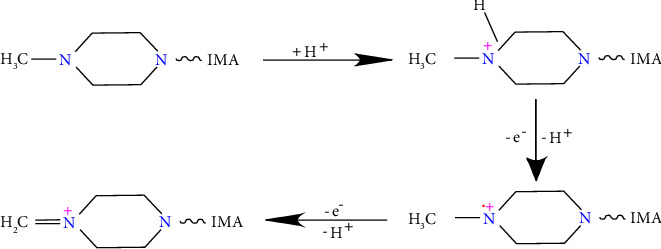
Possible mechanism for IMA on the AB/GCE.

In Figure 5(c), the peak current of IMA was linearly correlated with the scanning rate at 60–160 mV s^−1^, and the linear equation was *I* (*μ*A) = 0.279*v* − 4.864 (*R*^2^ = 0.983). The results indicated that the reaction system of IMA in this study was controlled by the surface. On this basis, the relationship between the logarithm of the scanning rate (ln*v*) and oxidation peak potential was discussed, and a linear equation of EP (*V*) = 0.028lnv + 0.689 (*R*^2^ = 0.997) was made as shown in Figure 5(d). This fully confirmed the above conclusion that the oxidation reaction of IMA on the prepared sensor was not reversible. In conclusion, IMA had a surface-controlled irreversible oxidation reaction on AB/GCE, and further analysis required the application of the following relationship [24]:

(2) $$\begin{array}{c} EP=\frac{E0+RT}{\left(\alpha nF\right)}\ln\left[\frac{RTk0}{\left(\alpha nF\right)}\right]+\frac{RT}{\left(\alpha nF\right)}\ln\;\nu , \end{array}$$

where E0 is the standard potential, *T* is the temperature, *α* and *F* represent the transfer coefficient and Faraday constant, and *k*0 and *n* represent the electron-transfer rate constant and electron-transfer number. We took an *α* value of 0.5 when an irreversible reaction occurred and then obtained *n* = 1.94. Therefore, the oxidation reaction of MTX at AB/GCE was a two-electron and one-proton electrochemical process, as can be seen in Scheme 1.

### 8.4. Verification

#### 8.4.1. Linearity, Sensitivity, and Precision Study

**Figure 6:**
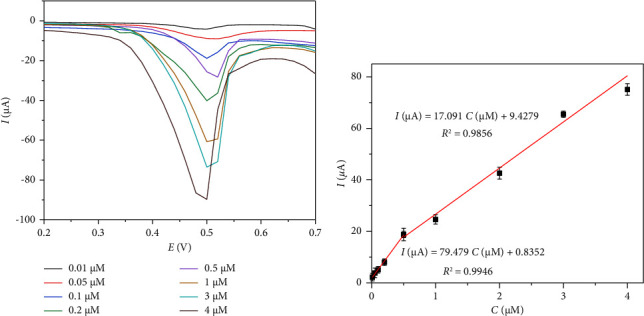
(a) DPV curves of different concentrations of IMA by the GCE/AB sensor. (b) Calibration curves of IMA under the optimized conditions.

The analytical measurement range obtained by DPV under optimal conditions is shown in Figure 6(a). The figure shows that the peak current values are directly proportional to the concentrations of IMA. After linear fitting, two linear ranges (Figure 6(b)) were found in the IMA concentration range of 0.01–4 *μ*M represented by *I* (*μ*A) = 79.476*C* (*μ*M) + 0.8352 (*R*^2^ = 0.9946) (0.01–0.5 *μ*M) and *I* (*μ*A) = 17.091*C* (*μ*M) + 9.4279 (*R*^2^ = 0.9856) (0.5–4 *μ*M). The limit of detection (LOD) of the method obtained from LOD = *kS*_*b*_/*m* was 0.15 nM [25], where *k* = 3, *S*_*b*_ is the standard deviation of the blank signals, and *m* is the slope of the calibration curve (the lower concentration range).

The performance of the proposed sensor was compared with that of the sensors in the literature, and the results are shown in Table 1. The finding shows that the GCE/AB sensor possesses wider linear ranges and a higher sensitivity supported by a lower LOD than the other sensors. Moreover, the GCE/AB sensor requires simple materials and preparation methods. Therefore, the preparation of the GCE/AB electrochemical sensor for IMA detection is necessary.

In addition, the precision of the prepared GCE/AB sensor was evaluated in this study. IMA at 1 *μ*M was repeatedly detected six times within one day, and the coefficient of variation (CV%) of the peak current values was as low as 6.52%. One electrode was prepared daily to detect IMA at 1 *μ*M one week, and the CV% of the peak current values was as low as 8.31%. The results show that the within-day precision and the day-to-day precision satisfy the IMA detection requirement. Therefore, the GCE/AB sensor has excellent reproducibility and stability for IMA detection.

#### 8.4.2. Accuracy

*(1) Interference Test*. Anti-interference capacity is an important factor that may affect the accuracy of IMA detection through electrochemical methods [30]. In this study, the effects of some organic and inorganic substances on IMA detection were investigated by adding different potential interfering substances to PBS containing 1 *μ*M of IMA. If the change in the peak current of IMA induced by the interfering substances is less than ±10%, then noninterference is recorded.

Table 2 provides the interference levels of several substances for the 1 *μ*M IMA detection. In the presence of 1000-fold of Na^+^, K^+^, and Cl^−^, 200-fold of Fe^3+^, Fe^2+^, Zn^2+^, and SO_4_^2−^, 100-fold of glucose, 50-fold of vitamin C and citric acid, 10-fold of glycine, and fivefold of folic acid, the potential substances imposed a noninterference effect on IMA detection. Thus, the GCE/AB sensor exhibited a satisfactory anti-interference capacity for IMA detection.

*(2) Addition Recovery Test*. An addition recovery test is used to assess the ability of experimental methods to accurately determine the pure analytes added to the conventional samples, and the results are expressed as recovery (%) [31]. Three concentration levels of IMA (0.1, 1, and 2 *μ*M) were added to healthy human serum, and then, the peak current values were detected by the GCE/AB sensor. The recovered concentrations were calculated according to the calibration curve of each added concentration, and the recovery (%) obtained is shown in Table 3.

The IMA recovery in this work was within the range of 90.1%–98.98%, and the relative standard deviation (RSD) was within the range of 0.3%–8.6%, indicating that the prepared GCE/AB electrochemical sensor can be used in the accurate detection of IMA.

## 9. Conclusions

In this research, a novel electrochemical sensor based on AB-modified GCE was successfully prepared for IMA determination. Within the allowable error limits, the sensor has a good linearity with ranges of 0.01–0.5 *μ*M and 0.5–4 *μ*M. In addition, the sensor exhibited high sensitivity with a low LOD of 0.15 nM and excellent precision. Moreover, the sensor displayed satisfactory anti-interference capacity and accuracy for IMA detection. Therefore, this study provides a practical platform for IMA determination, creating a new possibility for improving the usage safety of clinical drugs.

## Figures and Tables

**Table 1:** Analytical performance of different IMA electrochemical sensors.

Sensors	Linear range (*μ*M)	LOD (nM)	References
MWCNT-COOH SPCE	0.050–0.912	7.00	[26]
Fe_3_O_4_@MWCNTs@PANNFs	0.0017–0.85	0.40	[27]
NiO-ZnO/MWCNT-COOH/GCE	0.015–2.0	2.40	[28]
BDDE	0.03–0.25	6.30	[29]
GCE/AB	0.01–0.5, 0.5–4	0.15	This work

**Table 2:** Interference levels of several substances for detecting 1 *μ*M of IMA.

Substances	Interference level
K^+^, Na^+^, Cl^−^	1000
Zn^2+^, Fe^3+^, Fe^2+^, SO_4_^2−^	200
Glucose	100
Vitamin C, citric acid	50
Glycine	10
Folic acid	5

**Table 3:** IMA detection in the human serum by the GCE/AB sensor.

Serum	Added (*μ*M)	Found (*μ*M)	Recovery (%)	RSD (%) (*n* = 3)
1	0.1	0.0901	90.1	0.63
	1	0.9786	97.9	6.6
	2	1.9483	97.4	3.5

2	0.1	0.0906	90.62	1.9
	1	0.9021	90.22	8.6
	2	1.9797	98.98	1.1

3	0.1	0.0924	92.36	0.8
	1	0.9112	91.12	0.3
	2	1.9630	98.15	0.7

## Data Availability

The data used to support the findings of the study are available from the corresponding author upon request.
